# Association between dual use of e-cigarette and cigarette and chronic obstructive pulmonary disease: an analysis of a nationwide representative sample from 2013 to 2018

**DOI:** 10.1186/s12890-021-01590-8

**Published:** 2021-07-13

**Authors:** Taeyun Kim, Jihun Kang

**Affiliations:** 1 Division of Pulmonology, Department of Internal Medicine, The Armed Forces Goyang Hospital, Goyang-si, Republic of Korea; 2 Department of Family Medicine, Kosin University College of Medicine, Kosin University Gospel Hospital, 262 Gamcheon-ro, Seo-gu, Busan, Republic of Korea

**Keywords:** E-cigarette, COPD, Dual use

## Abstract

**Background:**

The association between the dual use of electronic cigarette (e-cigarette) and conventional cigarettes (c-cigarette) and spirometry-defined chronic obstructive pulmonary disease (COPD) has not been studied thoroughly.

**Methods:**

A total of 47,217 participants were identified in the 2013–2018 Korea National Health and Nutrition Examination Survey; of them, 12,919 participants aged ≥ 40 who underwent spirometry and had no missing data were enrolled. Pulmonary function testing, urinary cotinine, and urinary 4-(methylnitrosamino)-1-(3-pyridyl)-1-butanol (NNAL) levels were compared between dual users, current smokers, former smokers, and non-users using complex sample linear regression analysis. The odds ratio (OR) for COPD was calculated using a complex sample logistic regression model after adjusting for covariates.

**Results:**

Among current e-cigarette users, approximately 85% of the participants used c-cigarette concurrently, and 1.3% of all the participants were dual users (2.3% in males and 0.1% in females). Both dual users and current smokers showed higher levels of urine cotinine and NNAL than non-users and former smokers. The weighted prevalence of COPD was the highest in dual users (13.8% for all participants and 14.1% for males). The multivariate-adjusted OR of COPD for male dual users, compared to non-users, was 3.46 (*P*_trend_ < 0.001). The OR for COPD was 3.10 (*P*_trend_ < 0.001) in middle-aged (40–64 years) and 3.70 (*P*_trend_ < 0.001) in older (≥ 65 years) men. In females, the association was not observed and could not be precisely measured because of the small proportion of the smoking population.

**Conclusions:**

Dual use of e-cigarette and c-cigarette is associated with COPD in males.

**Supplementary Information:**

The online version contains supplementary material available at 10.1186/s12890-021-01590-8.

## Introduction

Chronic obstructive pulmonary disease (COPD) refers to a group of diseases that cause airflow limitation and breathing problems due to airway and/or alveolar damage [1]. COPD includes a spectrum of diseases that not only cause airway inflammation but also lead to several systemic consequences and comorbidities [2]. The Global Burden of Disease Study 2016 estimated that 251 million people had COPD, the fourth leading cause of death worldwide in those aged 50–74 years and the third leading cause in those aged > 75 in 2019 [3, 4]. The prevalence of COPD in South Korea among adults aged above 40 years is approximately 13%, which is slightly higher than the global prevalence of 12% [5, 6].

Electronic cigarette (e-cigarette) users inhale an aerosol that is produced by heating a liquid that usually delivers nicotine, flavorings, and other chemicals, and bystanders may also breathe in second-hand e-cigarette byproducts. In South Korea, the prevalence of e-cigarette use was approximately 4% among adolescents, and that of smoking was 7.8% in 2015 [7]. Although conventional smoking is a well-known cause of COPD [8], the association between vaping and the risk of COPD has not been established. Previous studies in the United States reported that e-cigarette users had a higher risk of chronic bronchitis, emphysema, and COPD than non-smokers [9]. This finding is further supported by experimental evidence suggesting that e-cigarette leads to airway inflammation. For example, exposure to e-cigarette liquid leads to increased reactivity, enlarged airspace, mucus hypersecretion, and increased expression of protease [10]. However, a recent study with a small sample size showed that switching to e-cigarette from conventional cigarette (c-cigarette) ameliorated pulmonary function decline and frequency of COPD exacerbation [11].

The difficulties associated with evaluating the effects of e-cigarettes on the risk of COPD have been reported in previous studies. First, because most e-cigarette users concurrently smoked c-cigarettes [12–15], comparing the association between e-cigarettes and COPD according to smoking status could be challenging. Although several biochemical methods using urine samples, such as cotinine and 4-(methylnitrosamino)-1-(3-pyridyl)-1-butanol (NNAL) have been introduced, limited progress has been made in the effort to differentiate e-cigarette users from cigarette users [16–18]. Second, individuals with COPD were determined using self-reported questionnaires rather than spirometry exam [9, 19, 20], therefore, the proportion of participants with COPD could be underestimated [21]. Third, the sample sizes were relatively small [19]. Fourth, potential confounders, such as height and social behaviors, were not measured or adjusted. Fifth, in Asia, few studies have been conducted on e-cigarette use and pulmonary function. Sixth, risk stratification between sexes may differ because the discrepancy in e-cigarette use by sex may differ. For example, in Hong Kong, a study suggested that e-cigarette use by males is much higher than by females [22]. Thus, examining the relationship between e-cigarette usage and COPD by appropriately considering other factors, such as smoking, diagnostic method, sample size, and sex disparity, has been important.

In this context, the present study aimed to evaluate the association between the dual use of e-cigarette and c-cigarette and COPD among Korean adults aged ≥ 40 who underwent spirometry using the Korea National Health and Nutritional Examination Survey (KNHANES).

## Materials and methods

### Study participants

The sixth and seventh KNHANES datasets (2013 to 2018) were used in this study. The study protocol for the survey has been described previously [23]. In brief, the KNHANES is a population-based, cross-sectional health and nutritional survey conducted annually by the Division of Chronic Disease Surveillance under the Korea Centers for Disease and Prevention and the Korean Ministry of Health and Welfare. A multistage, complex sampling method was used to represent non-institutionalized citizens of South Korea citizens. The KNHANES dataset is freely available on the website of the Korea Centers for Disease Control and Prevention.

**Fig. 1:**
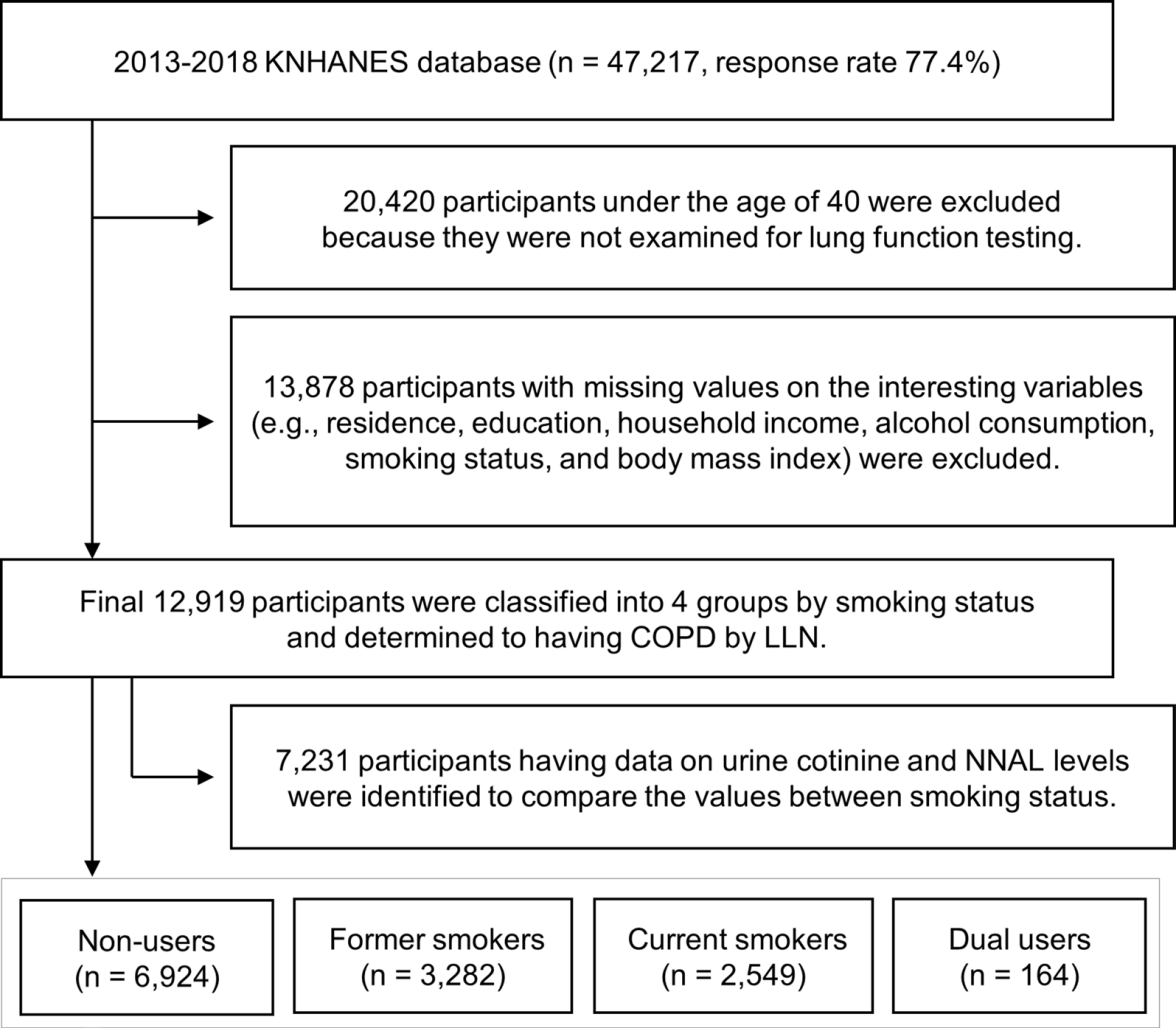
Flow diagram of the study participant selection process

A study flow chart of the process for selecting the participants is presented in Fig. 1. The sixth and seventh KNHANES assessed the health and nutritional status of 61,010 citizens, and 47,217 responded to the survey, with a response rate of 77.4%. Because pulmonary function testing (PFT) was conducted only in adults aged > 40 years, 20,420 adults were excluded. Subsequently, 13,878 adults with missing values were excluded. Finally, 12,919 adults were included in the analysis.

### Data measurements

Data regarding e-cigarette use were collected via self-reported questionnaires asking the following: (1) “Have you ever used an e-cigarette in your lifetime?” and (2) “Have you used an e-cigarette in the past 30 days?” Those who smoked less than 100 cigarettes in their lifetime or had never smoked and responded “no” to both questions were categorized as non-users. Those who had smoked more than 100 cigarettes in their lifetime but did not smoke currently and did not use an e-cigarette in the past 30 days were categorized as former smokers. Those who had smoked more than 100 cigarettes in their lifetime, smoke currently, and did not use an e-cigarette in the past 30 days were categorized as current smokers. Dual users were defined as those who smoke currently, have smoked more than 100 cigarettes in their lifetime, and used an e-cigarette in the past 30 days.

PFT was performed using dry rolling seal spirometers (Model 2130; SensorMedics, Yorba Linda, CA, USA) in 2013–2015 and Vyntus Spiro (CareFusion, San Diego, CA, USA) in 2016–2018. Quality control and standardization were conducted according to the criteria of the American Thoracic Society and the European Respiratory Society [24]. Forced expiratory volume in 1 s (FEV_1,_ Liter [L]), predicted FEV_1_%, forced vital capacity (FVC, L), predicted FVC%, and percentage of FEV_1_/FVC from the pro-bronchodilator test were measured.

Because the KNHANES did not evaluate the post-bronchodilator test data and because an FEV_1_/FVC ratio < 70% in the pre-bronchodilator test overestimates the prevalence of COPD [25], the lower limit of normal (LLN) was introduced to define COPD in the current study [26]. The equation used to estimate the LLN of FEV_1_/FVC in the Korean population has been previously described [27].

Urine cotinine levels (ng/mL) were determined by high-performance liquid chromatography-mass spectrometry (HPLC–MS) using API 4000 with an Agilent 1100 Series (AB Sciex, Framingham, MA, USA). Urinary 4-(methylnitrosamino)-1-(3-pyridyl)-1-butanol (NNAL) level (pg/mL) was determined by HPLC–MS using a Triple Quadrupole 5500 with Agilent 1200 Series (AB Sciex, Framingham, MA, USA). Urine cotinine and NNAL levels were used to compare values according to smoking status. Urine cotinine and NNAL levels were only available in the 2016–2018 KNHANES database.

Data on sociodemographic characteristics, including age, sex, residence, educational level, household income, and high-risk drinking were collected. Residences were categorized into rural and urban areas. Educational level was divided into three groups: middle school or lower, high school, and college or higher. Body mass index (BMI) was calculated as body weight (kg) divided by the squared height (m^2^). BMI was divided into three groups according to the Korean Society for the Study of Obesity guidelines [28]: < 23 kg/m^2^, 23–24.9 kg/m^2^, and ≥ 25 kg/m^2^. High-risk alcohol drinking was defined as seven (alcohol 60 g) or more drinks for men and five (alcohol 40 g) or more drinks for women on one occasion [29], and frequency of alcohol consumption was divided into > once per week or < once per week.

### Statistical analysis

The participants of the KNHANES were selected by proportional allocation system sampling with multistage stratification based on age, sex, and geographical area. Sampling weights were constructed for the study participants to represent the non-institutionalized resident population. Therefore, to prevent biased estimation and inordinate significance level [30], all analyses in this study were performed using complex sample analysis in SPSS, incorporating sample weights, stratification, and clustering of the KNHANES.

Categorical variables were compared using the chi-square test and presented as percentages and 95% confidence intervals (CIs). The distribution of urine cotinine and NNAL levels between smoking statuses were presented using boxplots, and statistical differences between groups were also calculated. The estimated values of lung function parameters according to smoking status were measured using complex sample linear regression analysis. A complex sample logistic regression model was used to calculate the odds ratio (OR) and 95% CI for COPD according to smoking status. In all the analyses, unweighted and weighted estimates were presented together. The *P*-values for trend were measured considering smoking status as a continuous variable in the model. We used a fully adjusted model that considered sex, residence, education, household income, alcohol consumption, and BMI as covariates.

All statistical analyses were performed using SPSS (version 24 for Windows, Chicago, USA). For all analyses, *P*-value < 0.05 was considered statistically significant.

## Results

Table 1 shows the general characteristics of the study participants. The participants were categorized into four groups: non-users (n = 6924, 53.6%), former smokers (n = 3282, 25.4%), current smokers (n = 2549, 19.7%), and dual users (n = 164, 1.3%). The percentages of all citizens in KNHANES and study participants selected according to e-cigarette and c-cigarette usage are shown in Additional file 1: Table S1 and S2, respectively. Of the current e-cigarette users, 84.6% used c-cigarettes concurrently, 12.3% smoked previously, and 1.2% never smoked. Because all never-smoked current e-cigarette users were aged < 40, they were not included in the analysis. The proportion of concurrent users was the highest in the 40–49-year age group. Dual users had the highest level of education. Compared to non-users, former smokers, and current smokers, dual users had the highest household income, alcohol consumption levels, and BMI (≥ 23).

**Table 1:** Characteristics of participants according to smoking status (N = 12,919)

	Non-users (n = 6924)	Former smokers (n = 3282)	Current smokers (n = 2549)	Dual users (n = 164)	*P*-value
Population size	54,021,767	28,005,146	23,324,272	1,632,492	
Men	19.2 (18.1–20.4)	93.6 (92.6–94.5)	89.8 (88.5–91.0)	95.0 (89.9–97.6)	< 0.001
*Age (years)*					< 0.001
40–49	38.2 (36.6–39.8)	28.8 (26.9–30.8)	46.2 (43.8–48.7)	68.9 (60.3–76.3)	
50–59	31.8 (30.5–33.1)	32.0 (30.0–34.0)	35.1 (32.8–37.4)	24.3 (17.5–32.5)	
60–69	19.7 (18.5–21.0)	22.8 (21.3–24.4)	13.0 (11.7–14.4)	3.2 (1.7–5.9)	
≥ 70	10.2 (9.4–11.2)	16.4 (15.0–17.8)	5.7 (4.8–6.8)	3.6 (1.5–8.7)	
*Residence*					0.031
Urban	85.1 (82.9–87.0)	83.7 (81.3–85.9)	82.4 (79.5–85.0)	86.9 (80.0–91.7)	
Rural	14.9 (13.0–17.1)	16.3 (14.1–18.7)	17.6 (15.0–20.5)	13.1 (8.3–20.0)	
*Education*					< 0.001
Middle school or lower	32.3 (30.7–33.9)	26.1 (24.2–28.1)	21.0 (19.2–23.0)	14.9 (9.3–23.2)	
High school	34.8 (33.3–36.3)	34.0 (32.0–36.1)	41.3 (39.1–43.7)	38.5 (30.1–47.7)	
College or more	32.9 (31.2–34.7)	39.9 (37.6–42.3)	37.6 (35.2–40.2)	46.5 (37.8–55.5)	
*Household income*					0.002
Lowest	13.9 (12.9–15.5)	14.8 (13.4–16.4)	13.5 (12.0–15.2)	6.1 (3.0–11.9)	
Lower middle	23.4 (22.1–24.7)	23.1 (21.5–24.9)	25.0 (23.0–27.1)	27.4 (19.9–36.4)	
Higher middle	27.6 (26.2–29.0)	27.1 (25.3–29.0)	30.7 (28.5–33.0)	34.1 (26.1–43.0)	
Highest	35.1 (33.4–36.9)	34.9 (32.7–37.2)	30.7 (28.4–33.2)	32.4 (24.6–41.3)	
*High-risk drinking*^*a*^					< 0.001
< 1/week	87.7 (86.7–88.6)	61.1 (59.1–63.1)	50.1 (47.8–52.4)	49.4 (40.7–58.1)	
≥ 1/week	12.3 (11.4–13.3)	38.9 (36.9–40.9)	49.9 (47.6–52.2)	50.6 (41.9–59.3)	
*BMI (kg/m*^*2*^*)*				< 0.001	
< 23	41.3 (39.8–42.7)	29.2 (27.5–31.0)	34.9 (32.7–37.1)	26.6 (19.6–35.0)	
23–24.9	25.4 (24.2–26.6)	28.4 (26.7–30.2)	27.1 (25.0–29.3)	29.7 (22.6–38.0)	
≥ 25	33.3 (32.0–34.7)	42.4 (40.4–44.4)	38.0 (35.8–40.3)	43.7 (35.0–52.8)	
Urine cotinine (ng/mL)	17.0 (11.8–22.1)	44.5 (29.2–59.9)	1320.5 (1259.3–1381.7)	1617.9 (1391.8–1844.2)	< 0.001
Urine NNAL (pg/mL)	4.4 (2.3–6.4)	5.1 (3.8–6.3)	190.9 (171.7–209.9)	187.3 (134.2–240.3)	< 0.001

Data are presented as weighted percentages with 95% confidence intervals for categorical variables, unless otherwise stated

Comparisons of categorical variables from complex sample surveys were performed using the chi-squared test

BMI, body mass index; NNAL, 4-(methylnitrosamino)-1-(3-pyridyl)-1-butanol

^a^High-risk drinking was defined as seven or more drinks for men and five or more drinks for women on one occasion

The associations between e-cigarette and cigarette smoking status and pulmonary function testing (PFT) are presented in Table 2. Predicted FEV_1_% and FEV_1_/FVC% showed a negative association with smoking status. Overall, predicted FEV_1_% and FEV_1_/FVC% were the lowest in dual users and the highest in non-users, and this trend was retained for men. However, no significant association between smoking status and predicted FEV_1_% was observed in the women. There was no significant association between predicted FVC% and smoking status.

**Table 2:** Pulmonary function testing by smoking status in Korean adults

	FEV_1_, Liter/sec	Predicted FEV_1_, %	FVC, Liter/sec	Predicted FVC, %	FEV_1_/FVC, %
*Total (N* = *12,919)*					
Non-users (n = 6924)	2.61 (2.58–2.63)	90.3 (89.6–91.0)	3.39 (3.36–3.42)	90.5 (89.9–91.2)	76.8 (76.4–77.2)
Former smokers (n = 3282)	2.58 (2.55–2.61)	88.7 (87.9–89.6)	3.44 (3.40–3.47)	90.5 (89.8–91.2)	75.3 (74.9–75.8)
Current smokers (n = 2549)	2.56 (2.53–2.59)	87.9 (87.1–88.8)	3.45 (3.42–3.49)	91.1 (90.4–91.9)	74.5 (74.1–75.0)
Dual users (n = 164)	2.55 (2.45–2.64)	86.4 (84.1–88.7)	3.45 (3.33–3.56)	90.0 (87.8–92.1)	74.2 (72.9–75.5)
*P*_trend_	< 0.001	< 0.001	0.007	0.215	< 0.001
*Men (n* = *6596)*					
Non-users (n = 1165)	3.01 (2.96–3.06)	89.7 (88.4–91.0)	3.94 (3.89–4.00)	89.2 (88.2–90.3)	76.0 (75.3–76.7)
Former smokers (n = 3039)	2.98 (2.95–3.02)	87.8 (86.7–88.9)	3.99 (3.95–4.04)	89.1 (88.3–89.9)	74.4 (73.7–75.0)
Current smokers (n = 2237)	2.93 (2.89–2.97)	86.4 (85.2–87.6)	3.99 (3.94–4.03)	89.4 (88.5–90.3)	73.1 (72.5–73.8))
Dual users (n = 155)	2.90 (2.80–3.01)	85.1 (82.6–87.6)	3.97 (3.85–4.09)	88.2 (86.0–90.5)	72.8 (71.5–74.2)
*P*_trend_	< 0.001	< 0.001	0.250	0.927	< 0.001
*Women (n* = *6323)*					
Non-users (n = 5759)	2.23 (2.21–2.26)	91.5 (90.6–92.4)	2.85 (2.82–2.88)	92.3 (91.5–93.1)	78.1 (77.7–78.5)
Former smokers (n = 243)	2.25 (2.19–2.31)	90.5 (88.4–92.6)	2.91 (2.84–2.98)	92.7 (90.8–94.6)	77.1 (76.1–78.0)
Current smokers (n = 312)	2.28 (2.23–2.33)	91.2 (89.6–92.9)	2.95 (2.88–3.02)	93.5 (91.9–95.1)	77.0 (76.3–77.8)
Dual users (n = 9)	2.14 (1.78–2.50)	88.0 (76.6–99.4)	2.85 (2.48–3.22)	92.4 (83.3–101.5)	74.7 (69.4–80.0)
*P*_trend_	0.144	0.420	0.003	0.153	< 0.001

Values of pulmonary function testing with 95% confidence intervals were calculated after adjustments for sex, age, residence, education, household income, alcohol consumption, and body mass index

The *P*-value for trend was calculated using complex sample logistic regression analysis considering smoking status as a continuous variable

FEV_1_, forced expiratory volume in one second; FVC, forced vital capacity

**Fig. 2:**
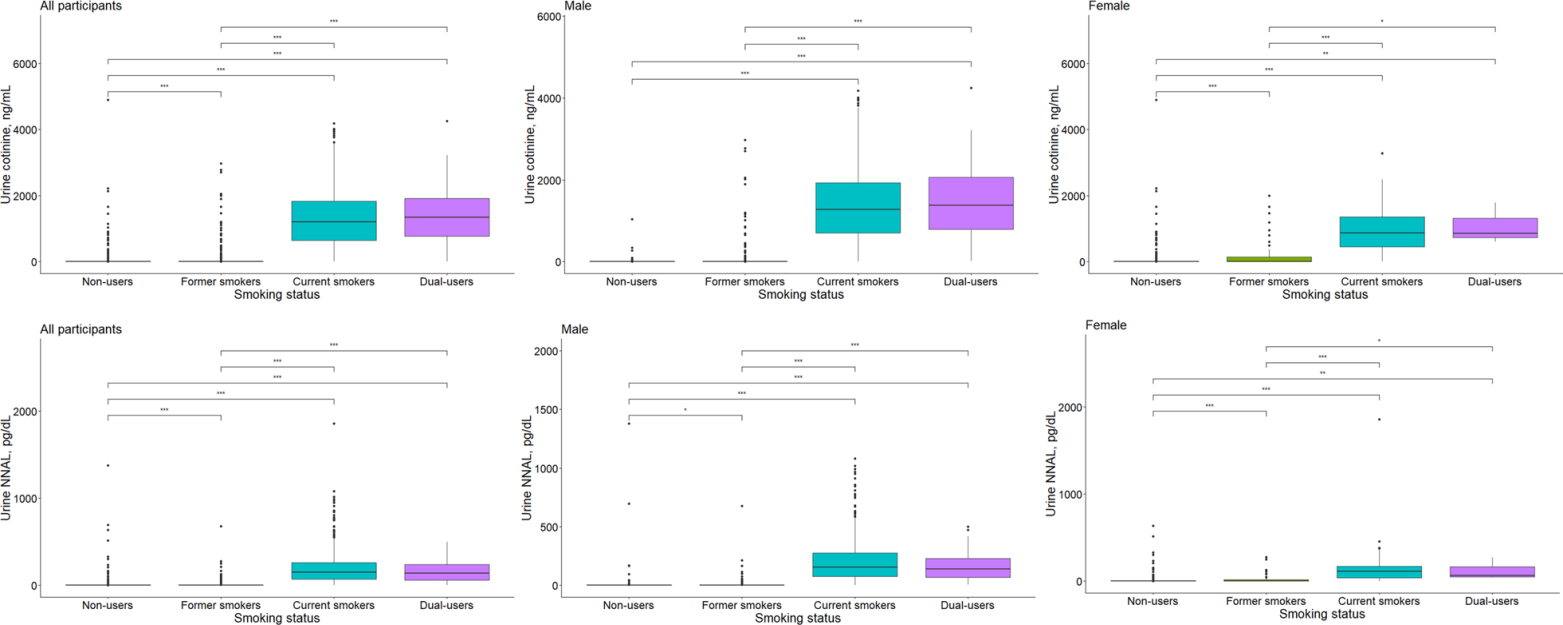
Comparison of urinary cotinine and 4-(methylnitrosamino)-1-(3-pyridyl)-1-butanol (NNAL) levels according to electronic cigarette exposure by sex

Both current smokers and dual users presented higher levels of urine NNAL and cotinine levels than non-users and former smokers (Table1 and Fig. 2). However, no differences in urine NNAL and cotinine levels were observed between current smokers and dual users.

The weighted estimate of the prevalence of COPD was 8.8% in Korean adults (Table 3). The percentage was higher for men (10.8%) than for women (6.4%). A total of 164 (1.3%) adults used c-cigarette and e-cigarette concurrently (155 [2.3%] men and 9 [0.1%] women). Among the men, the OR for COPD was 3.46 (95% CI: 1.89–6.34) in dual users, 2.69 (95% CI: 1.93–3.75) in current smokers, and 1.81 (95% CI: 1.31–2.49) in former smokers, compared to in non-users (*P*_trend_ < 0.001).

**Table 3:** The prevalence and OR for COPD by smoking status in the Korean adults

	Prevalence (%, 95% CI)	OR (95% CI)	*P*-value
*Total (n* = *12,919)*	8.8 (8.3–9.4)		
Non-users (n = 6924)	6.0 (5.4–6.6)	1	
Former smokers (n = 3282)	10.7 (9.5–12.1)	1.67 (1.31–2.12)	< 0.001
Current smokers (n = 2549)	12.9 (11.4–14.6)	2.26 (1.77–2.88)	< 0.001
Dual users (n = 164)	13.8 (8.9–20.7)	2.83 (1.64–4.86)	< 0.001
*P*_trend_	< 0.001	< 0.001*	
*Men (n* = *6596)*	10.8 (10.0–11.8)		
Non-users (n = 1165)	5.7 (4.4–7.5)	1	
Former smokers (n = 3039)	10.8 (9.5–12.2)	1.81 (1.31–2.49)	< 0.001
Current smokers (n = 2237)	13.2 (11.6–15.0)	2.69 (1.93–3.75)	< 0.001
Dual users (n = 155)	14.1 (9.0–21.3)	3.46 (1.89–6.34)	< 0.001
*P*_trend_	< 0.001	< 0.001*	
*Women (n* = *6323)*	6.4 (5.7–7.1)		
Non-users (n = 5759)	6.0 (5.4–6.7)	1	
Former smokers (n = 243)	9.7 (6.1–15.0)	1.62 (0.96–2.74)	0.059
Current smokers (n = 312)	10.1 (6.9–14.5)	1.47 (0.93–2.34)	0.100
Dual users (n = 9)	7.8 (0.9–39.2)	1.09 (0.14–8.48)	0.950
*P*_trend_	< 0.001	0.038*	

Considering that the Korea National Health and Nutrition Examination Survey is a multi-stage clustered probability design, weighted percentages and 95% CIs were presented

Odds ratios and 95% CIs were calculated after adjustment for sex, age, residence, education, household income, alcohol consumption, and body mass index

COPD, chronic obstructive pulmonary disease; OR, odds ratio; CI, confidence interval

*P-value for trend was calculated using complex sample logistic regression analysis considering smoking status as a continuous variable

The results of subgroup analyses involving middle-aged (40–64 years) and older (≥ 65 years) adults are shown in Table 4. For all participants, the weighted estimate of the prevalence of COPD was 7.8% in middle-aged adults and 13.1% in older adults. The prevalence was 9.2% in middle-aged men and 17.2% in older men. Considering dual use, the prevalence was 13.2% in middle-aged men and 28.0% in older men. In middle-aged men, the adjusted OR for COPD was the highest in dual users, i.e., 3.10 (95% CI: 1.55–6.21), followed by current smokers, former smokers, and non-users. The ORs of COPD for older men were 3.70 (95% CI: 0.96–14.23) in dual users, 2.94 (95% CI: 1.68–5.15) in current smokers, and 1.93 (95% CI: 1.21–3.09) in former smokers, compared to in non-users.

**Table 4:** The prevalence and OR for COPD by smoking status in the middle-aged (40–64 years) and older (65 and more) Korean adults

	Prevalence (%, 95% CI)	OR (95% CI)	*P*-value	Prevalence (%, 95% CI)	OR (95% CI)	*P*-value
	Aged 40–64 (n = 9896)			Aged 65 and more (n = 3023)		
*Total*	7.8 (7.2–8.5)			13.1 (11.8–14.7)		
Non-users	5.6 (5.0–6.3)	1		7.4 (6.0–9.1)	1	
Former smokers	8.3 (7.1–9.8)	1.49 (1.11–2.00)	0.008	17.0 (14.5–19.7)	2.12 (1.38–3.25)	0.030
Current smokers	11.5 (9.9–13.3)	1.97 (1.50–2.60)	< 0.001	24.4 (19.5–30.2)	3.27 (1.99–5.39)	< 0.001
Dual users	12.9 (7.9–20.3)	2.45 (1.35–4.46)	0.003	28.0 (10.2–57.1)	4.14 (1.11–15.39)	0.030
*P*_trend_	< 0.001	< 0.001*		< 0.001	< 0.001*	
*Men*	9.2 (8.3–10.2)			17.2 (15.2–19.4)		
Non-users	4.6 (3.2–6.5)	1		10.2 (6.7–15.2)	1	
Former smokers	8.3 (7.0–9.9)	1.82 (1.19–2.79)	0.006	17.0 (14.5–19.8)	1.93 (1.21–3.09)	0.008
Current smokers	11.9 (10.2–13.8)	2.55 (1.70–3.85)	< 0.001	24.0 (18.9–30.0)	2.94 (1.68–5.15)	< 0.001
Dual users	13.2 (8.0–21.0)	3.10 (1.55–6.21)	0.001	28.0 (10.2–57.1)	3.70 (0.96–14.23)	0.050
*P*_trend_	< 0.001	< 0.001*		< 0.001	< 0.001*	
*Women*	6.1 (5.4–6.9)			7.5 (6.0–9.3)		
Non-users	5.9 (5.2–6.7)	1		6.6 (5.2–8.5)	1	
Former smokers	8.6 (4.9–14.7)	1.50 (0.78–2.73)	0.236	16.0 (7.9–29.8)	2.35 (0.99–5.58)	0.002
Current smokers	8.2 (5.2–12.7)	1.13 (0.66–1.95)	0.649	29.0 (13.8–50.9)	4.74 (1.76–12.73)	0.051
Dual users	7.2 (0.9–39.2)	0.94 (0.12–7.13)	0.948	–	–	–
*P*_trend_	< 0.001	0.444*		< 0.001	0.001*	

Considering that the Korea National Health and Nutrition Examination Survey is a multi-stage clustered probability design, weighted percentages and 95% CIs were presented

Odds ratios and 95% CIs were calculated after adjustment for age, sex, residence, education, household income, alcohol consumption, and body mass index

COPD, chronic obstructive pulmonary disease; OR, odds ratio; CI, confidence interval

*P-value for trend was calculated using complex sample logistic regression analysis considering smoking status as a continuous variable

## Discussion

The present cross-sectional study evaluated the association between dual use of e-cigarette and c-cigarette and COPD using a nationally representative sample of Korean adults. Dual use of e-cigarettes and c-cigarette was negatively associated with predicted FEV_1_% and FEV_1_/FVC%, especially in Korean males. In addition, in the male population, the OR for COPD defined by LLN was the highest for dual users, followed by current smokers and former smokers, compared to non-users. This association was more prominent in older (65 years and older) adults than in middle-aged (40–64 years) adults. To our knowledge, this is the first Asian study to evaluate the association between dual use and COPD. Although the current study did not evaluate the sole effect of e-cigarettes on COPD, understanding the additive effects of e-cigarettes in the context of disease burden could be important, and the results of this study might be noteworthy and warrant further longitudinal studies.

Although cigarette smoking is a risk factor for COPD [8], the role of e-cigarette in the development of COPD has not been widely evaluated, especially in Asian populations. In line with the current study, a recent cross-sectional study in the United States that included more than 700,000 participants and 14,036 dual users showed that the use of e-cigarette was associated with an increased risk of COPD (OR: 1.64 [95% CI 1.34–2.00]) [20]. In addition, a 2-year follow-up study on the association between e-cigarette use and incident respiratory diseases (i.e., COPD, chronic bronchitis, emphysema, and asthma) indicated that e-cigarette is an independent risk factor for obstructive lung diseases [9]. The Population Assessment of Tobacco and Health study also revealed that e-cigarette was associated with a high prevalence of self-reported COPD [19]. However, despite the large sample sizes in previous studies, COPD was defined based on self-reported or telephone surveys; thus, the misclassification of COPD might, at least in part, have affected the magnitude of association in those studies.

The dual use of e-cigarette and c-cigarette is important in public health because e-cigarette has been marketed as a less harmful substitute for c-cigarette and a large number of individuals, especially young adults, use e-cigarette and c-cigarette concurrently [31]. The estimate of the prevalence of current e-cigarette use from the Behavioral Risk Factor Surveillance System in 2016 was 4.5% [31]. The overall percentage of dual users in our study was 1.3%, which was slightly lower than that in previous studies from the United States (1.8% to 2.0%) [20, 31] and New Zealand (2.7%) [12]. Importantly, among current e-cigarette users, 84.6% used e-cigarette and c-cigarette concurrently in the present study, and the rate of concurrent use was comparable with the rates reported in other countries: 74% in Germany [13], 83% in France [15], 65% in New Zealand [12], and 76% in Japan [14]. Considering that most e-cigarette users also use c-cigarette, it is challenging to determine the independent effect of e-cigarette on the risk of COPD.

The present study found a stepped increase in the OR for COPD, with the highest OR for dual users. Smoking status in the current study was categorized into four groups using self-reported questionnaires, and urine cotinine and NNAL levels were measured. Both urine cotinine and NNAL levels were higher in dual users and current smokers than in former smokers and non-users. This difference was observed in both men and women. Current smokers showed higher levels of both urine cotinine and NNAL levels than former smokers and non-smokers [17]. Considering that the KNHANES questionnaire distinguished former smokers from current smokers, if the participants did not smoke within a year and considering the half-lives of NNAL (10–16 days) and cotinine (16 h) [32, 33], the smoking statuses reported in the present study were well categorized and the observed association seems to be supported by biochemical data. In addition, since e-cigarette use does not modify urine NNAL levels significantly [16], the difference in the value between dual users and current smokers might be minimal. Moreover, because of the heterogeneity in the use of e-cigarette, determining the cut-off values for exposure to e-cigarette using urine cotinine and NNAL levels has been challenging [18]. Correspondingly, the current study estimated the association of e-cigarette with COPD in the context of dual use of e-cigarette and c-cigarette, rather than the sole use of e-cigarette.

Interestingly, in the current study, older male dual users presented a higher OR for COPD than those aged 40–64 years. However, no interaction effect between age and smoking status was observed (data not shown). The prevalence of e-cigarette use is lower in older adults than in middle-aged adults [34]. There are several explanations for this finding. First, age is an independent risk factor for COPD [1], and aging could make the lung vulnerable to tissue damage from several toxicants of e-cigarettes and susceptible to COPD development [35]. Second, many people in South Korea have tuberculosis, which is also a risk factor for COPD [36], and the prevalence of tuberculosis is high in older adults [37].

Given that the lung is the primary site of entrance for several chemical components in e-cigarette, acute or chronic pulmonary toxicities could be provoked. The levels of innate defense proteins, such as elastase and matrix metalloproteinase-9, which are closely related to COPD, were significantly elevated in induced sputum samples from e-cigarette users [38]. A mouse model highlighted that inhalation of nicotine-containing e-cigarette liquid led to airway hyper-reactivity, mucin hypersecretion, and cytokine and protease expression, collectively resulting in lung tissue destruction [10]. Redox imbalance caused by increased production of pro-inflammatory cytokines and decreased lung glutathione levels results in oxidative stress and inflammatory response in the lung, which are the key pathologic features of COPD [39]. Furthermore, exposure to e-cigarette could negatively affect anti-microbial defense, making the lungs susceptible to infection [40]. These pulmonary toxicities related to e-cigarette are similar to those of c-cigarette smoking and challenge the concept that e-cigarette is less harmful than c-cigarette [38].

Several chemical components of e-cigarette may be linked to lung injury. First, nicotine is the most commonly used chemical substance. An increase in the secretion of interleukin-6 and 8 by human airway epithelial cells was observed in mice exposed to nicotine-containing e-cigarette liquid [10]. Mice exposed to e-cigarette liquid without nicotine did not show a significant change in cytokine expression [10]. Exposure to nicotine-containing e-cigarettes impairs proteostasis and autophagy, suggesting the role of e-cigarette in the pathogenesis of emphysema [41]. Second, some toxicants, such as diacetyl and acetyl propionyl, are present at higher levels in e-cigarette than in c-cigarette [42]. Propylene glycol (PG) and vegetable glycerol (VG) are the vaporizing solvents commonly used in e-cigarettes. PG and VG disturb the glucose transport function in the airway epithelium and decrease glucose metabolism, which collectively contribute to airway damage when repeated and chronic exposure to e-cigarette occurs [43]. Acrolein, a thermal byproduct of PG and VG, is connected to COPD by its role in altering intracellular signaling, oxidative stress, mucus hypersecretion, and protease-mediated airway damage [44].

The present study defined COPD using an LLN level of the lower fifth percentile of the reference population. Using a fixed ratio of FEV_1_/FVC of < 0.7 to classify participants as having COPD is a common method in epidemiologic studies. However, using the fixed ratio alone may lead to an overestimation of COPD [45]. An FEV_1_/FVC values of < 0.7 may be found in up to 20% of healthy subjects aged over 60 years [46]. It could be better to diagnose airflow obstruction based on the frequency distribution of the reference population using appropriate cutoffs in large sample surveys [26, 46]. In addition, several epidemiological studies have classified participants as having COPD based on self-reported surveys [9, 19, 20]. COPD patients are likely to overreport themselves as former smokers or current e-cigarette users [20]. Recall bias also occurs when remembering smoking habits and the clinical diagnosis of COPD [19].

Our study has several limitations. First, although our study found a negative association between e-cigarette and COPD, the results should be interpreted cautiously, as they are derived from cross-sectional survey data and do not provide causality. Second, we did not estimate figures pertaining to switching to e-cigarette from c-cigarette; thus, if former or current smokers recently chose e-cigarette as a smoking-cessation strategy, this might have affected the observed association. Third, because other smoking-related residual confounders such as pack-year, years quit smoking, types of e-cigarette delivery vehicles, flavors of e-cigarette, and consumption pattern (daily or intermittent) were not included in the analysis owing to the unavailability of the data in KNHANES, the observed findings may have been altered. Fourth, females comprise a very small part of the smoking and e-cigarette population. Given that sex influences the risk factors for COPD, risks may differ in females. Fifth, considering the self-reported nature of the KNHANES, recall bias in smoking status should be taken into account for.

Despite these limitations, the results of this study are noteworthy as they reveal the potentially harmful effect of dual use of e-cigarette and c-cigarette on lung function. Moreover, given that the Korean Ministry of Health and Welfare recommend citizens not to use flavored e-cigarette and prohibited teenagers from using the product in October 2019 and the Constitutional Court rejected the petition of the Korea Electronic Cigarette Association against its constitutionality in March 2020, the results of the current study may influence the establishment of policies regarding e-cigarettes.

## Conclusion

Dual use of e-cigarette and c-cigarette is associated with an increased risk of COPD, as defined by the lower fifth percentile of a healthy reference group in the male population. The magnitude of the association was strong in older men aged > 65 years. Although switching to e-cigarette from c-cigarette theoretically reduces the harm from smoking, concurrent use might result in a synergistic negative effect on lung function, and appropriate policymaking should be based on solid evidence for e-cigarette use.

## Supplementary Information

**Additional file 1. Table S1:** Percentage of all Korea National Health and Nutrition Examination Survey participants (*N* = 34,241, age ≥19 years) according to electronic(e)-cigarette use and smoking status. **Table S2:** Percentage of eligible study participants (*N* = 12,919, age ≥40 years) according to electronic(e)-cigarette and smoking status.

## Data Availability

Data cannot be made publicly available by the authors, because they were obtained from the 2013–2018 Korea National Health and Nutrition Examination Surveys (KNHANES) data. All files are available from the KNHANES webpage (URL: https://knhanes.cdc.go.kr/knhanes/index.do). The authors confirm that future interested researchers will be able to obtain and access KNHANES data in the same manner as the authors of this manuscript. The authors confirm that they did not have any special assess privileges that others would not have.
