# Supplementary material for: Association between dual use of e-cigarette and cigarette and chronic obstructive pulmonary disease: an analysis of a nationwide representative sample from 2013 to 2018
Source: BMC Pulm Med. 2021 Jul 13;21:231. doi: 10.1186/s12890-021-01590-8 (PMC8278700; doi:10.1186/s12890-021-01590-8)
Supplement: Supplementary file 1 — Additional file 1. Table S1: Percentage of all Korea National Health and Nutrition Examination Survey participants (N = 34,241, age ≥19 years) according to electronic(e)-cigarette use and smoking status. Table S2: Percentage of eligible study participants (N = 12,919, age ≥40 years) according to electronic(e)-cigarette and smoking status. [file 12890_2021_1590_MOESM1_ESM.docx]

**Supplementary Table 1**. Percentage of all Korea National Health and Nutrition Examination Survey participants (N = 34,241, age ≥19 years) according to electronic(e)-cigarette use and smoking status

| Conventional cigarette use | | E-cigarette | | | Total |
| --- | --- | --- | --- | --- | --- |
|  |  | Never | Ever* | Current |  |
| Non-users | Frequency | 21377 | 51 | 20 | 21448 |
|  | Percentage of non-users | 99.7% | 0.2% | 0.1% | 100.0% |
|  | Percentage of e-cigarette users | 66.9% | 3.1% | 3.1% | 62.6% |
| Former | Frequency | 6061 | 354 | 78 | 6493 |
|  | Percentage of former smokers | 93.3% | 5.5% | 1.2% | 100.0% |
|  | Percentage of e-cigarette users | 19.0% | 21.2% | 12.3% | 19.0% |
| Current | Frequency | 4498 | 1264 | 538 | 6300 |
|  | Percentage of current smokers | 71.4% | 20.1% | 8.5% | 100.0% |
|  | Percentage of e-cigarette users | 14.1% | 75.7% | 84.6% | 18.4% |
| Total | Frequency | 31936 | 1669 | 636 | 34241 |
|  | Percentage of smokers | 93.3% | 4.9% | 1.9% | 100.0% |
|  | Percentage of e-cigarette users | 100.0% | 100.0% | 100.0% | 100.0% |

* Participants who had used e-cigarettes in their lifetime but did not use any within the past 30 days were classified as ever e-cigarette users.

**Supplementary Table 2**. Percentage of eligible study participants (N = 12,919, age ≥40 years) according to electronic(e)-cigarette and smoking status

| Conventional cigarette use | | E-cigarette | | | Total |
| --- | --- | --- | --- | --- | --- |
|  |  | Never | Ever* | Current |  |
| Non-users | Frequency | 6915 | 9 | 0 | 6924 |
|  | Percentage of non-users | 99.9% | 0.1% | 0.0% | 100.0% |
|  | Percentage of e-cigarette users | 56.6% | 1.7% | 0.0% | 53.6% |
| Former | Frequency | 3167 | 115 | 0 | 3282 |
|  | Percentage of former smokers | 96.5% | 3.5% | 0.0% | 100.0% |
|  | Percentage of e-cigarette users | 25.9% | 21.2% | 0.0% | 25.4% |
| Current | Frequency | 2131 | 418 | 164 | 2713 |
|  | Percentage of current smokers | 78.5% | 15.4% | 6.0% | 100.0% |
|  | Percentage of e-cigarette users | 17.4% | 77.1% | 100.0% | 21.0% |
| Total | Frequency | 12213 | 542 | 164 | 12919 |
|  | Percentage of smokers | 94.5% | 4.2% | 1.3% | 100.0% |
|  | Percentage of e-cigarette users | 100.0% | 100.0% | 100.0% | 100.0% |

* Participants who had used e-cigarettes in their lifetime but did not use any within the past 30 days were classified as ever e-cigarette users.
